# The CRP troponin test (CTT) stratifies mortality risk in patients with non‐ST elevation myocardial infarction (NSTEMI)

**DOI:** 10.1002/clc.24256

**Published:** 2024-03-28

**Authors:** Rafael Y. Brzezinski, Shmuel Banai, Malka Katz Shalhav, Moshe Stark, Ilana Goldiner, Ori Rogowski, Itzhak Shapira, David Zeltser, Noa Sasson, Shlomo Berliner, Yacov Shacham

**Affiliations:** ^1^ Internal Medicine “C” and “E”, Tel Aviv Sourasky Medical Center, Affiliated with the Faculty of Medicine Tel Aviv University Tel Aviv Israel; ^2^ Department of Cardiology, Tel Aviv Sourasky Medical Center, Affiliated with the Faculty of Medicine Tel Aviv University Tel Aviv Israel; ^3^ Department of Emergency Medicine, Tel Aviv Sourasky Medical Center, Affiliated with the Faculty of Medicine Tel Aviv University Tel Aviv Israel; ^4^ Division of Clinical Laboratories, Tel Aviv Sourasky Medical Center, Affiliated with the Faculty of Medicine Tel Aviv University Tel Aviv Israel

**Keywords:** acute coronary syndrome, CRP, inflammation, NSTEMI, troponin

## Abstract

**Introduction:**

The C‐reactive protein (CRP)‐troponin‐test (CTT) comprises simultaneous serial measurements of CRP and cardiac troponin and might reflect the systemic inflammatory response in patients with acute coronary syndrome. We sought to test its ability to stratify the short‐ and long‐term mortality risk in patients with non‐ST elevation myocardial infarction (NSTEMI).

**Methods:**

We examined 1,675 patients diagnosed with NSTEMI on discharge who had at least two successive measurements of combined CRP and cardiac troponin within 48 h of admission. A tree classifier model determined which measurements and cutoffs could be used to best predict mortality during a median follow‐up of 3 years [IQR 1.8–4.3].

**Results:**

Patients with high CRP levels ( > 90th percentile, >54 mg/L) had a higher 30‐day mortality rate regardless of their troponin test findings (16.7% vs. 2.9%, *p* < 0.01). However, among patients with “normal” CRP levels ( < 54 mg/L), those who had high troponin levels ( > 80th percentile, 4,918 ng/L) had a higher 30‐day mortality rate than patients with normal CRP and troponin concentrations (7% vs. 2%, *p* < 0.01). The CTT test result was an independent predictor for overall mortality even after adjusting for age, sex, and comorbidities (HR = 2.28 [95% CI 1.56‐3.37], *p* < 0.01 for patients with high troponin and high CRP levels).

**Conclusions:**

Early serial CTT results may stratify mortality risk in patients with NSTEMI, especially those with “normal” CRP levels. The CTT could potentially assess the impact of inflammation during myocardial necrosis on the outcomes of patients with NSTEMI and identify patients who could benefit from novel anti‐inflammatory therapies.

## Graphical abstract

We examined 1,675 patients diagnosed with NSTEMI who had at least two successive measurements of CRP and cardiac troponin within 48 h of admission. A tree classifier model determined which cutoffs could be used to best predict mortality. The CRP‐Troponin‐Test (CTT) might identify inflammatory‐prone patients with worse outcomes following NSTEMI.

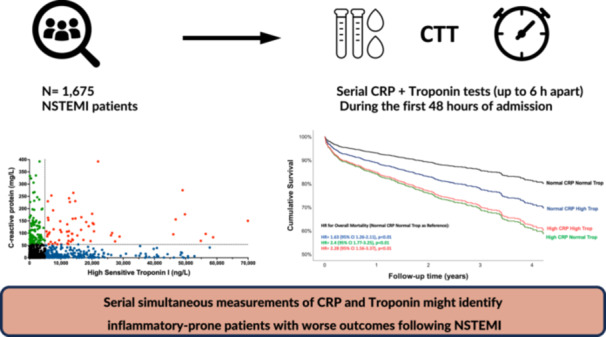

## 1 INTRODUCTION

Elevated cardiac troponin is a hallmark of non‐ST elevation myocardial infarction (NSTEMI).^1^ The definition of NSTEMI includes several clinical entities, such as type 1 MI, type 2 MI, and myocardial injury.^2^ Distinguishing between these entities is challenging, and differentiating between them bears substantial implications on patient management and prognosis.^3^, ^4^, ^5^ Thus, definitive diagnostic tests aimed at better stratifying risk at the onset of NSTEMI development are needed.

A rapid increase in C‐reactive protein (CRP) concentrations early in the development of acute coronary syndrome (ACS) is associated with worse outcomes and cardiac dysfunction.^6^, ^7^, ^8^, ^9^, ^10^, ^11^, ^12^, ^13^ We recently described a simple diagnostic approach for identifying STEMI patients at increased risk for worse outcomes, the “CRP Troponin Test (CTT)”, which is comprised of simultaneous serial measurements of both CRP and cardiac troponin during admission.^14^, ^15^ However, the clinical application of this approach in patients with NSTEMI has not yet been studied.

Systemic and vascular inflammation play a key role in the development of ACS in addition to precipitating illnesses, such as infection, respiratory disease, and kidney injury.^16^, ^17^, ^18^ CRP is an acute phase reactant. Analyzing its concentrations in relation to circulating cardiac troponin, which implicates myocardial necrosis, might help identify “inflammatory prone” patients who demonstrate an excessive inflammatory response during an MI.

We, therefore, sought to determine the association between early CTT results to short‐ and long‐term mortality rates in patients with NSTEMI. We also aimed to characterize patients according to their inflammatory response as measured by the CTT during the first 48 h of admission.

## 2 MATERIALS AND METHODS

### 2.1 Study design and clinical data

We performed a retrospective observational study at the Tel‐Aviv Sourasky Medical Center. We included consecutive adult patients ( > 18 years of age) admitted to the emergency room (ER) or cardiac intensive care unit (CICU) who were ultimately diagnosed with acute NSTEMI on discharge between January 2017 and April 2022. Only patients who had at least two successive measurements of both CRP and cardiac troponin levels within the first 48 h of admission were included in the final study cohort (72% of patients). The CTT result was derived from test findings of a CRP and a troponin measurement carried out with an up to 6 h gap between them. The diagnosis of NSTEMI was based on a clinical presentation of chest pain, diagnostic electrocardiographic changes, and serial elevation of serum cardiac biomarkers.^1^

The MDClone platform was used to automatically extract multiple demographic, clinical, and laboratory variables from electronic health records as well as determine 30‐day and overall mortality rates.^19^ We adjusted for pre‐existing comorbidities by means of the Charlson comorbidity index.^20^ The median follow‐up duration for overall mortality was 3 years (interquartile range [IQR] 1.8–4.3).

Wide‐range CRP (wr‐CRP) levels were determined by the Siemens wr‐CRP assay (Siemens Healthcare diagnostic Inc NY, USA)^21^ whose measurement range is 0.03–160 mg/L.^22^ High‐sensitivity cardiac troponin I was measured by an ADVIA Centaur® TNIH assay (Siemens Healthcare). The TNIH assay measures cTnI concentrations from 2.50 to 25,000.00 ng/L.

### 2.2 Statistical analysis

All continuous variables are displayed as means ( ± standard deviation [SD]) for normally distributed variables or median [IQR] for variables with abnormal distribution. Categorical variables are displayed as numbers (%) of subjects within each group. Continuous variables were compared by a Student's t‐test for normally distributed variables and by the Mann‐Whitney U test or Kruskal‐Wallis test for non‐normally distributed ones. Related samples were compared by a Wilcoxon Signed Rank test. To assess associations among categorical variables, we used a chi‐square test. We assessed normal distributions with Kolmogorov–Smirnov's test and Q–Q plots.

To determine which CRP and troponin measurements (baseline or serial) and corresponding cut‐offs should be used to predict overall mortality we used Chi‐square automatic interaction detection (CHAID).^23^ The CHAID analysis builds a predictive model to determine the best cutoffs for the input variables for predicting an outcome. In CHAID, the continuous predictors are split into categories with an approximately equal number of observations. CHAID creates all possible cross‐tabulations for each categorical predictor until the best outcome has been achieved and no further splitting can be performed.

The patients were divided into four groups according to the output cutoffs. Thirty‐day mortality rates were evaluated using binary logistic regression. Overall mortality was evaluated by means of univariate and multivariable Cox proportional hazard regression. Cumulative survival curves divided by CTT cutoff groups were derived. We adjusted our models for the combined second CTT test finding, age, sex, and Charlson comorbidity index score. The median follow‐up duration for measuring overall mortality was calculated using the reverse Kaplan‐Meir method. A two‐tailed *p* value < .05 was considered statistically significant. All analyses were performed with the SPSS (IBM SPSS Statistics, version 28, IBM Corp., Armonk, NY, USA, 2016), the R statistical package version 3.6.1 (R Foundation for Statistical Computing, Vienna, Austria), and the GraphPad Prism version 9.00 (GraphPad Software, La Jolla, CA, USA).

## 3 RESULTS

### 3.1 Patient characteristics

**Figure 1:**
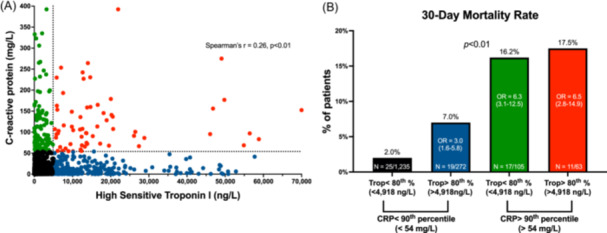
The CRP troponin test (CTT) and 30‐day mortality. (A) A scatterplot of the study population according to their second CRP and cardiac troponin test results. The dotted lines represent the 90th and 80th percentile thresholds (CRP > 54 mg/L and cardiac troponin >4918 ng/L, respectively) according to our CHIAD analysis as described in the Methods section. (B) Bar graphs representing the proportion of patients who died during 30 days since admission. *p*‐values were calculated by the chi‐square test. We performed binary logistic regression to predict 30‐day mortality and adjusted our model for the combined CTT test status, age, sex, and the Charlson comorbidity index score. *OR* ‐ Odds ratios (95% confidence interval) are presented inside the bar graphs with the group of patients who had CRP and cardiac troponin levels below the 90th and 80th percentiles as reference.

The final study cohort included 1,675 patients with NSTEMI. Patient characteristics according to the CTT cutoff groups are displayed in Table 1. The cohort's mean age ± SD was 70.6 ±  13.6 years, and 498 patients (30%) were females. The entire cohort's first and second troponin levels were 235 [73‐1,219] and 930 [219–3,534] ng/L, *p* < 0.01 for median [IQR]. Their CRP levels increased modestly from 5.7 [1.7–14.8] to 6.3 [1.7–16.3] mg/L, *p* < 0.01 for median [IQR]. The distribution of the second CTT test results and the correlation between CRP and cardiac troponin is presented in Figure 1A and Supp Figure 1. A total of 747 patients (44.6%) had a second CRP ≤ 5 mg/L, which is considered the upper normal limit of the assay.

**Table 1:** Patient characteristics according to C‐reactive protein troponin test results.

	CRP <90th %ile ( < 54 mg/L)		CRP >90th %ile ( > 54 mg/L)		
Characteristic	Trop <80th %ile ( < 4918 ng/L)	Trop >80th %ile ( > 4918 ng/L)	Trop <80th %ile ( < 4918 ng/L)	Trop >80th %ile ( > 4918 ng/L)	*p*
N (total = 1675)	1235	272	105	63	
Age, yrs ( ± SD)	69.4 (13.7)	72.4 (13.3)	76.8 (10.6)	76.9 (11.7)	<0.01
Females, *n* (%)	369 (30)	79 (29)	36 (34)	14 (22)	0.42
BMI, kg/m^2^ ( ± SD)	27.1 (4.5)	27.2 (4.5)	26.7 (5.1)	27.0 (4.5)	0.83
Comorbidities					
Hyperlipidemia, n (%)	512 (41)	113 (42)	34 (32)	27 (43)	0.33
Hypertension, n (%)	596 (48)	134 (49)	59 (56)	40 (63)	0.06
Diabetes, n (%)	440 (36)	108 (40)	59 (56)	33 (52)	<0.01
Past MI, n (%)	96 (8)	29 (11)	14 (13)	4 (7)	0.12
Past CVA, n (%)	123 (10)	30 (11)	20 (19)	9 (15)	0.03
Heart failure, n (%)	122 (10)	32 (12)	21 (20)	8 (14)	0.01
Renal disease, n (%)	136 (11)	53 (20)	35 (34)	22 (37)	<0.01
Peripheral vascular disease, n (%)	78 (6)	27 (10)	20 (19)	12 (20)	<0.01
Autoimmune disease, n (%)	35 (3)	8 (3)	3 (3)	3 (5)	0.853
Charlson comorbidity index [IQR]	4.0 [2,5]	4.0 [2,6]	6.0 [4,7]	6.0 [4,8]	<0.01
Charlson comorbidity index 10‐year survival, % ( ± SD)	53.3 (37.6)	45.5 (37.5)	22.8 (29.9)	26.4 (32.9)	<0.01
Medications					
Hyperlipidemia, n (%)	634 (51)	133 (49)	69 (66)	33 (52)	0.03
Diabetes, n (%)	376 (30)	87 (32)	40 (38)	28 (44)	0.06
Antihypertension, n (%)	508 (41)	112 (41)	68 (65)	31 (49)	<0.01
Anticoagulants, n (%)	520 (42)	122 (45)	62 (59)	29 (46)	0.01
Antibiotics during admission, n (%)	151 (12)	54 (20)	77 (73)	41 (65)	<0.01
Laboratory					
Hemoglobin, g/L ( ± SD)	13.6 (1.9)	13.5 (2.1)	11.8 (2.4)	11.8 (2.0)	<0.01
WBC, 10^9^/L ( ± SD)	9.6 (3.3)	11.0 (3.7)	12.5 (5.0)	12.2 (4.1)	<0.01
Platelets, 10^3^/mcL	229 (78)	230 (73)	239 (82)	236 (81)	0.54
HbA1C, % [IQR]	6.0 [5.6, 6.8]	6.0 [5.5, 7.0]	6.6 [5.9, 7.9]	6.3 [5.9, 7.0]	0.01
Glucose, mg/dL ( ± SD)	151.0 (77.6)	172.7 (97.5)	187.0 (121.4)	187.0 (85.5)	<0.01
Creatinine, mg/dL [IQR]	1.0 [0.8, 1.2]	1.1 [0.8, 1.4]	1.3 [0.9, 2.3]	1.4 [1.0, 2.1]	<0.01
Total cholesterol, mg/dL ( ± SD)	173.4 (53.1)	175.0 (46.7)	141.7 (43.7)	167.2 (58.4)	<0.01
LDL cholesterol, mg/dL ( ± SD)	105.0 (47.3)	105.2 (42.3)	76.4 (34.2)	95.3 (40.0)	0.01
HDL cholesterol, mg/dL ( ± SD)	41.6 (13.0)	43.5 (14.5)	39.2 (11.9)	42.7 (14.8)	0.22
Triglycerides, mg/dL ( ± SD)	146.2 (127.5)	138.8 (93.9)	126.7 (57.1)	125.7 (72.2)	0.432
1st CRP, mg/L [IQR]	4.3 [1.4,10.4]	6.3 [1.7,14]	90.8 [66.7,129.9]	83.3 [53.1,145.4]	<0.01
2nd CRP, mg/L [IQR]	4.5 [1.4,10.8]	7.9 [2,16.6]	93.1 [74.5,142]	104.6 [72.6,156.1]	<0.01
1st troponin, ng/L [IQR])	145 [57,512]	2772 [659,6936]	692 [123,1731]	8489 [3873,14337]	<0.01
2nd troponin, ng/L [IQR]	458 [161,1519]	10119 [6816,17468]	1475 [480,2903]	13559 [8942,20226]	<0.01

Abbreviations: CTT, CRP troponin test (second measurement); CRP, C‐reactive protein; HbA1c, hemoglobin A1c; HDL, high‐density lipoprotein; LDL, low‐density lipoprotein; MI, myocardial infarction; Trop, cardiac troponin I; WBC, white blood count.

A total of 382 patients (22.8%) died during a median follow‐up period of 3 years [1.8–4.3]. We used a CHAID tree analysis to determine which CTT measurement and cutoffs should be used to predict overall mortality. A second CRP level >54 mg/L (90th percentile of the cohort) combined with a second troponin level >4918 ng/L (80th percentile) were associated with the highest overall mortality rate. Accordingly, patients were divided into four groups based on these cutoffs (Figure 1A and Table 1).

Patients with a high CRP level ( > 54 mg/L) were older and had a higher prevalence of comorbidities regardless of their troponin test result (Table 1). These patients had a higher Charlson comorbidity index score together with a higher prevalence of hypertension and diabetes. Of note, body mass index levels, as well as the prevalence of dyslipidemia were similar across the four groups. Moreover, low‐density lipoprotein cholesterol and total cholesterol levels were lower among patients with high CRP levels compared with patients with “normal” CRP levels (i.e., <54 mg/L), regardless of their troponin test result. Thus, high CRP levels were not necessarily associated with obesity (Table 1). Patients with high CRP levels also received more antibiotic therapy during admission compared to the rest of the cohort (Table 1).

### 3.2 CTT results and overall mortality

We first compared short‐ and long‐term mortality rates across the cohort according to their CTT test results. Patients with high CRP levels had a higher 30‐day mortality rate regardless of their troponin test status (16.7% vs. 2.9% for the rest of the cohort, *p* < .01) (Figure 1B). However, patients with “normal” CRP levels who had high troponin levels (above the 80th percentile [4918 ng/L]) had a higher 30‐day mortality rate compared with patients with “normal” CRP and troponin concentrations (7% vs. 2%, respectively, *p* < 0.01 (Figure 1B). CRP was associated with a higher 30‐day mortality rate also when analyzed as a continuous variable (OR = 1.12 (95% CI 1.09–1.16) per 10‐unit increase in CRP, *p* < 0.01).

**Figure 2:**
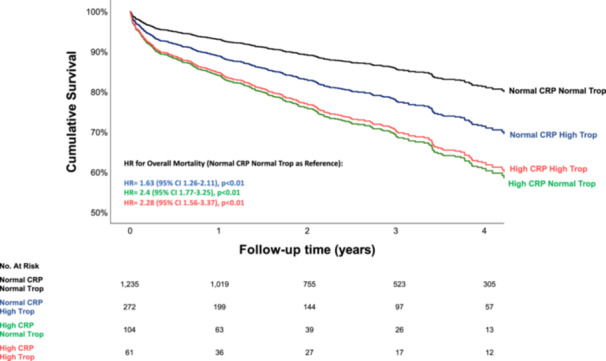
The CRP troponin rest (CTT) and long‐term mortality. Cox regression survival curves according to CTT test results. We adjusted our model for the combined CTT test status, age, sex, and the Charlson comorbidity index score. The bar and line colors correspond to the four groups presented in Figure 1. Adjusted hazard ratios for overall mortality and 95% confidence interval are presented for each group.

Long‐term overall mortality rates demonstrated a similar pattern. Survival curves and hazard ratios (HR) are presented in Figure 2. High CRP levels were associated with increased mortality rates regardless of troponin test status (Figure 2). However, patients with high troponin levels had higher overall mortality rates compared with patients with both “normal” CRP and “normal” troponin results (HR = 1.63 [95% CI 1.26‐2.11], *p* < 0.01). In summary, the CTT was able to stratify patients according to their overall mortality risk, especially those whose CRP levels were not extremely high ( < 54 mg/L). Finally, a multivariate Cox regression analysis showed that the second CTT test result was an independent predictor of overall mortality even after adjusting for age, sex, and comorbidities (Figure 2 and Table 2). CRP was associated with increased mortality rates also when analyzed as a continuous variable (HR = 1.07 (95% CI 1.06–1.08) per 10‐unit increase in CRP, *p* < 0.01).

**Table 2:** Multivariate Cox regression analysis to predict overall mortality.

		95% CI		
	Odds ratio	Lower	Upper	*p*
Age (years)	1.05	1.03	1.06	<0.01
Sex (female)	1.14	0.92	1.42	0.24
Charlson Comorbidity Index	1.25	1.19	1.31	<0.01
2nd CTT result (normal CRP and troponin as indicator)				
High troponin‐normal CRP	1.63	1.26	2.11	<0.01
Normal troponin‐high CRP	2.4	1.77	3.25	<0.01
High troponin‐high CRP	2.28	1.56	3.37	<0.01

Method = Enter.

Abbreviations: CI,confidence interval; CTT,CRP Troponin Test (second measurement); CRP, C‐reactive protein.

### 3.3 Referral to coronary angiography according to CTT test results

**Figure 3:**
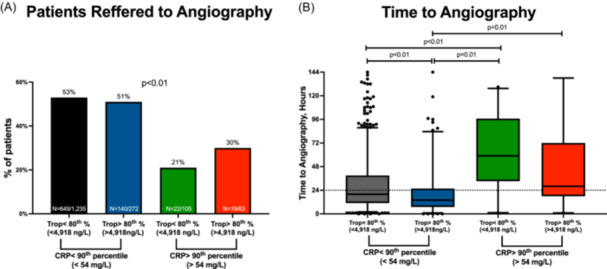
Combined CRP and troponin test (CTT) results and coronary angiography. (A) Bar graphs representing the proportion of patients who underwent coronary angiography. *p*‐values were calculated by the chi‐square test. (B) Boxplots representing the time from the patient's arrival to the emergency room until a coronary angiography was performed. *p*‐values were calculated by the Kruskal‐Wallis test with Dunn's test for multiple comparisons.

Next, we examined how many patients were ultimately referred to coronary angiography and how many hours had passed from their arrival at the ER until the procedure. A total of 830 patients (49.6%) underwent angiography during admission. The median time to angiography was 19.5 h [IQR 11–39]. Fewer patients with elevated CRP levels underwent angiography regardless of their troponin test status (24.4% vs. 52.4% of the patients with normal CRP levels, *p* < 0.01) (Figure 3). Specifically, only 19 patients (30%) with both high CRP and high troponin levels underwent angiography compared with 649 patients (53%) of patients with “normal” CRP and “normal” troponin results (*p* < 0.01). Patients with high CRP but “normal” troponin results ( < 4,918 ng/L) were the least likely to undergo angiography (*n* = 22 (21%)).

Furthermore, there were significant differences in time to angiography between the four groups. Patients with high CRP levels had longer intervals between admission and treatment (Figure 3). Patients with high troponin levels but relatively “normal” CRP results were the most rapidly catheterized, with a median time to angiography of 14 h [IQR 7‐25], *p* < 0.01. Notably, most patients with relatively “normal” CRP levels underwent angiography within the first 24 h of admission, while patients with high CRP levels did not (median time to angiography 19 [11–38] vs 41 [21–77], respectively, *p* < 0.01). Finally, a total of 582 patients who represent 70% of all patients referred to angiography were ultimately treated with a coronary intervention. The proportion of those patients was similar across the four CTT cutoff groups (70.1%, 71.4%, 72.7%, and 57.9%, *p* = 0.67.

## 4 DISCUSSION

The main finding of our study is that early serial CTT results during the first 48 h of admission may stratify the mortality risk in patients with NSTEMI, especially in those presenting with relatively “normal” CRP levels. Patients with extremely high CRP levels ( > 54 mg/L, 90th percentile of the current cohort) were at increased risk for 30‐day and overall mortality during a median follow‐up period of 3 years [1.8–4.3], regardless of their troponin test results. However, in patients with relatively “normal” CRP ( < 54 mg/L), those who had extremely high troponin concentrations ( > 4,918, 80th percentile of the cohort) had higher mortality rates than those with “normal” CRP and troponin concentrations.

We recently demonstrated the use of the CTT in patients with STEMI.^14^, ^15^ Unlike our current results, the CTT in the setting of STEMI was able to stratify mortality risk even among patients with extremely high CRP levels ( > 90th percentile). Nonetheless, those patients had higher mortality rates than patients with “normal” CRP levels, similar to the findings of this current report of NSTEMI patients. Taken together, our findings demonstrate that the CTT is a simple diagnostic tool that could potentially assess the impact of inflammation on the outcomes of patients with ACS during myocardial necrosis and thus improve their early risk stratification.

This study has several limitations. First, its retrospective design makes it susceptible to residual confounding. Second, the cutoffs for both the CRP and troponin levels were optimized for this specific study sample and may not reflect the same diagnostic yield in other populations. Moreover, the diagnosis of NSTEMI on discharge encompasses a highly heterogeneous study population, including patients with coexisting non‐coronary precipitating events.^24^ We are aware that these events, such as infection, decompensated heart failure, cardiac arrhythmias, anemia, uncontrolled hypertension, acute noninfectious respiratory disease, pericardial disease, and pulmonary embolism have a substantial effect on CRP and troponin kinetics and probably also on the relation between the two. However, it should be noted that the CTT was an independent predictor for overall mortality even after adjusting for age and comorbidities (Figure 2). We propose that the CTT could aid in clinical decision‐making, which often presents a challenge in terms of risk stratification and the need for an invasive approach, especially in these patients. We call for future large‐scale studies to examine the effect of these coexisting non‐coronary precipitating events on the dynamics of the CTT and its prognostic accuracy.

Finally, we show that most patients with high CRP levels in our cohort were not referred for coronary angiography (Figure 3). Moreover, even when they had been referred, angiography was not performed during the first 24 h from admission as it had been for most patients with “normal״ CRP levels (Figure 3). This, however, is in line with previous reports on the proportion of NSTEMI patients who undergo angiography during admission.^24^, ^25^ The relatively low rate of angiography performance and the longer time periods until the procedure was administered could be explained by the fact that the patients with high CRP levels were older and had more comorbidities and probably also higher rates of the precipitating events listed above. We hypothesize that these patients with elevated CRP levels are more “inflammatory prone” and might benefit from early anti‐inflammatory therapy, such as colchicine [12] and anti‐IL‐1ß agents [30], in addition to or instead of coronary intervention. The early use of the CTT could help identify these patients and help guide clinicians in their initial management.

In summary, we suggest that the CTT might be used to identify patients with an exaggerated inflammatory response to myocardial necrosis. This simple diagnostic tool could help identify patients at risk for worse outcomes who might benefit from novel anti‐inflammatory therapies following myocardial infarction or injury.

## AUTHOR CONTRIBUTIONS

RYB, YS, SB, and SB participated in study conception and design. RYB, NS, MK, YS, DZ, MS, and IG performed the acquisition of data. RYB and SB participated in analysis and interpretation of data. RYB, SB, and SB drafted the manuscript and OR, DZ, IS, YS, and SB helped in critical review of the manuscript. All of the authors have read and approved the submitted manuscript.

## CONFLICT OF INTEREST STATEMENT

The authors report no relationships that could be construed as a conflict of interest.

## Supporting information

Supplementary Figure 1. CRP and cardiac troponin distributions. Histograms of the second CRP (A) and cardiac troponin (B) test results.

## Data Availability

The data that support the findings of this study are available on request from the corresponding author. The data are not publicly available due to privacy or ethical restrictions.
