# Supplementary figures and images for: The CRP troponin test (CTT) stratifies mortality risk in patients with non‐ST elevation myocardial infarction (NSTEMI)
Source: Clin Cardiol. 2024 Mar 28;47(4):e24256. doi: 10.1002/clc.24256 (PMC10976426; doi:10.1002/clc.24256)

**A.**

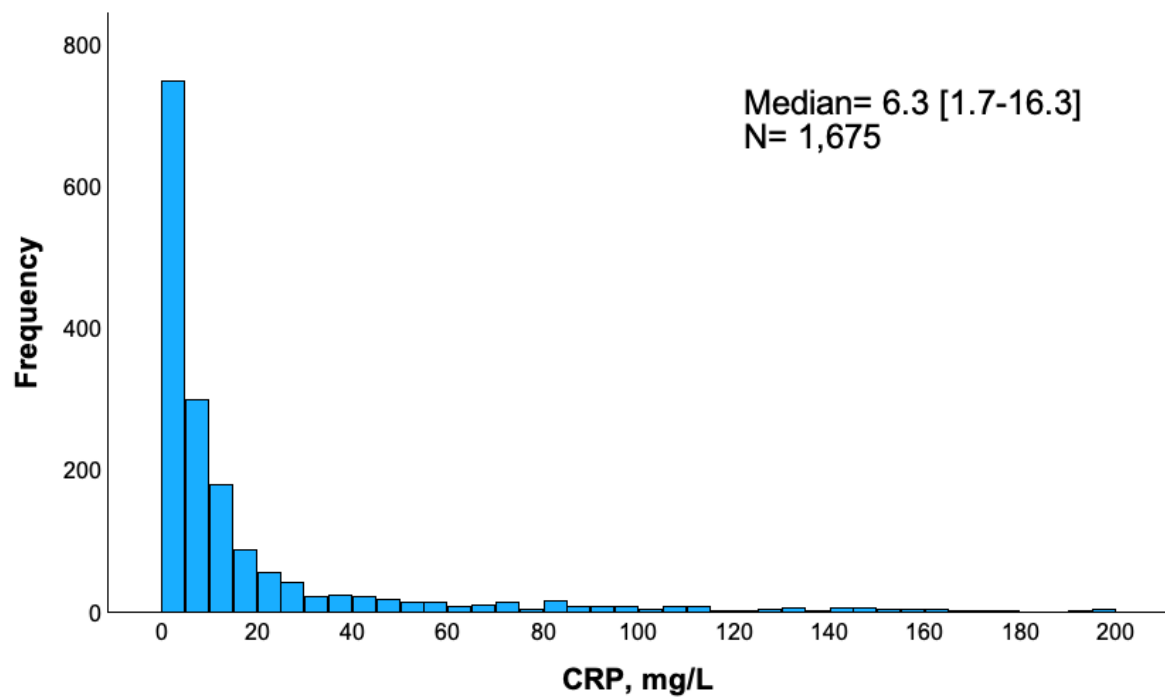

**B.**

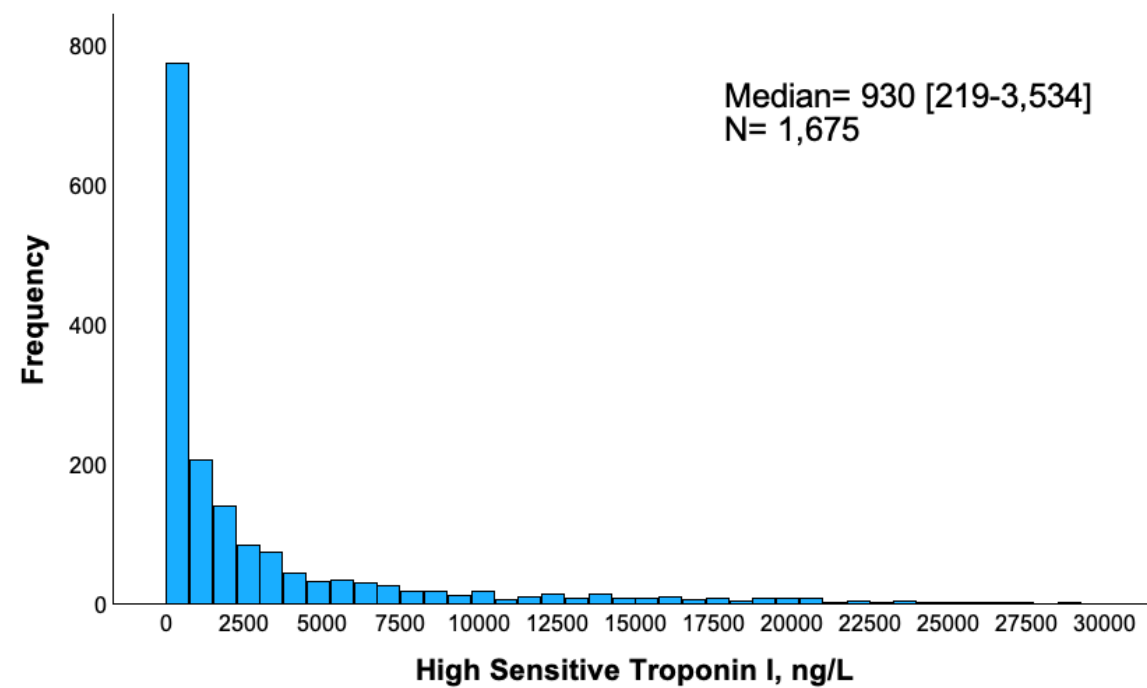

Supplement: Supplementary file 1 — Supplementary Figure 1. CRP and cardiac troponin distributions. Histograms of the second CRP (A) and cardiac troponin (B) test results. [file CLC-47-e24256-s001.pdf]
